# Functional Biomaterials for Regenerative Dentistry

**DOI:** 10.3390/jfb16080298

**Published:** 2025-08-19

**Authors:** Nicholas G. Fischer

**Affiliations:** MDRCBB-Minnesota Dental Research Center for Biomaterials and Biomechanics, University of Minnesota, 515 Delaware St. SE, Minneapolis, MN 55455, USA; fisc0456@umn.edu

## 1. Introduction

Regenerative dentistry hopes to restore oral health by replacing diseased or damaged tissues with biologically functional, integrated counterparts. This differs critically from past approaches that rely on bonded and inert synthetic materials. The oral cavity contains a complex array of tissues frequently affected by disease, including the periodontal ligament, the dental pulp, the alveolar bone, and the mineralized tooth itself. The regeneration of each of these components presents significant scientific and clinical hurdles. Addressing these challenges is crucial for achieving oral health and improving patients’ quality of life.

In this evolving landscape, biomaterials that stimulate and support tissue regeneration are slowly becoming indispensable tools in everyday dental practice. These materials’ potential is derived from their ability to be tailored to individual patients, their compatibility with living tissues, and their capacity to modulate immune responses—qualities that position regenerative biomaterials as the cornerstone of future dental therapies. Here, we highlight work from a Special Issue showcasing cutting-edge research on functional biomaterials for regenerative dentistry.

## 2. Special Issue Highlights

Medication-related osteonecrosis of the jaw (MRONJ) is a destructive bone condition caused by certain drugs, such as antiresorptive and antiangiogenic drugs, in the absence of radiation treatment. Minimal definitive treatment options exist. Advanced platelet-rich fibrin (A-PRF) is a platelet-rich blood preparation with high concentrations of growth factors that has been suggested as a possible treatment option for MRONJ. Adamska et al. [1] retrospectively studied clinical data from 28 patients presenting with osteomyelitis due to MRONJ. The authors determined that less advanced lesions, in the absence of risk factors, had a better prognosis than advanced lesions and that A-PRF showed no significant effect on MRONJ resolution.

A-PRF has been evaluated in a variety of ever-expanding oral maxillofacial surgery applications. As a result, Chmielewski et al. [2] performed a meta-analysis focused on the question, “Does A-PRF provide better clinical outcomes than other materials used in exact oral and maxillo-facial procedures?” The results generally showed some benefit in a variety of clinical procedures, such as bone healing and postoperative pain reduction, albeit with limited evidence. Further analysis by Chmielewski et al. [3] focused on advanced platelet-rich fibrin + (A-PRF+). In comparison to A-PRF, A-PRF+ is produced with a different centrifugation protocol and is supposed to have a higher concentration of growth factors and a different fibrin network structure. The authors’ systematic review showed that the performance of A-PRF+ was not significantly improved compared to other blood preparations, such as A-PRF, but there were slight trends toward improvement.

Decades of research have intensively investigated bone grafting materials. Dentin has been suggested as an alternative material given it may be autologous and is compositionally similar to bone. However, many dentin processing and preparation parameters remain poorly optimized. Mazzucchi et al. [4] examined the influence of tooth age on the dentinal release of growth factors important to bone growth. The authors showed that age did not affect growth factor release, albeit under the specific preparation parameters the authors used.

Clinicians frequently chose materials based on personal preferences and experiences. Friedmann et al. [5] compared three dental restorative centers that used either cross-linked high-molecular-weight hyaluronic acid (xHyA) with a xenograft, or enamel matrix derivative (EMD) with an allograft, or xHyA with a resorbable membrane for restoration of intrabody defects. The authors showed that, depending on the outcome variables, results were similar between groups, lending support to the use of xHyA compared to the classic EMD approach. Similarly, Panda et al. [6] compared plasma rich in growth factors (PRGFs) versus xenogenic bone graft (BXG) in patients for the regeneration of intrabony defects and showed similar results. The authors wisely noted “treatment decisions [should be] guided by patient-specific factors and clinical goals.”

Park et al. [7] explored the idea of an oral probiotic, *Weissella cibaria*, for potentially protecting against tooth-supporting bone destruction from periodontal diseases. Periodontal diseases are common and marked, as they advance, by osteoclast-mediated bone destruction. The authors showed in vitro that *Weissella cibaria* suppressed osteoclast differentiation and activity. Further research on delivery methods may enable further deployment of probiotics in regenerative dentistry.

Dental caries are one of the most widespread infectious diseases in the world. Consistent evidence for decades has supported the widespread implementation of fluoride. Continual refinement of fluoride-containing dental materials has been motivated by patient preferences. Iwawaki et al. [8] evaluated a high-concentration fluoride varnish in comparison to a typical fluoride mouthwash in an early enamel caries in vitro model and showed the high-concentration fluoride varnish did not increase remineralization of the subsurface demineralized layer, but the varnish did improve the acid resistance of the model carious lesion.

The rising popularity of ceramic veneers as indirect esthetic restorations has fueled intense interest in the development of longer-lasting restorative materials. In particular, the wear resistance of dental materials in the highly biologically and mechanically active oral cavity has been historically evaluated to ensure long-term, esthetic results. Oshika et al. [9] showed the wear resistance of light-curable resin luting cements was similar to dual-cure cements and flowable resin-based composites.

Dentistry has been at the forefront of nanotechnology with the advances in filler technologies for dental resin composites. Nanoparticles have been explored for a variety of applications, especially those nanoparticles composed of gold. Jongrungsomran et al. [10] reviewed the use of gold nanoparticles in dentistry and concluded there was substantial promise for their use, particularly for “enhancing biological, mechanical and optical properties.”

## 3. Concluding Remarks

By bringing together innovations in material science, biology, and clinical practice, regenerative dentistry is poised to redefine the standard of dental, oral, and craniofacial care. The contributions in this Special Issue advance the scientific understanding of functional biomaterials and accelerate their translation into routine dental procedures. As the boundary between restoration and regeneration continues to blur, the future of dentistry will be shaped by therapies that are not only reparative but truly regenerative—restoring both form and function in a patient-centered manner.
